# A meta-analysis of active smoking and risk of meningioma

**DOI:** 10.18332/tid/133704

**Published:** 2021-05-10

**Authors:** Hong Chao, Yu Cheng, Jie Shan, Hai-Feng Xue, Wei-Lan Xu, Hong-Jie Li, E Meng

**Affiliations:** 1 Public Health College, Qiqihar Medical University, Qiqihar, China; 2 The Third Affiliated Hospital, Qiqihar Medical University, Qiqihar, China; 3 International Education College, Qiqihar Medical University, Qiqihar, China; 4 Yangzhou Center for Disease Control and Prevention, Yangzhou, China

**Keywords:** meningioma, smoking, meta-analysis

## Abstract

**INTRODUCTION:**

Cigarette smoking has been hypothesized to be a risk factor for meningioma. However, the results of studies exploring the relationship between smoking exposure and the occurrence of meningioma are inconsistent.

**METHODS:**

A search of PubMed, Medline, Embase, and Science Direct (up to June 2020) databases was performed. Two authors independently extracted the data. The Newcastle–Ottawa Scale was employed for judging the quality of articles. A random-effects model was utilized for meta-analysis. Association analysis between smoking and meningioma was based on the adjusted RR and the 95% CI, as reported by eligible studies. Subgroup and sensitivity analyses were performed and publication bias was assessed. Subgroup analysis was conducted by geographical region, study design, sex, study quality, and adjustments of RR score. Begg’s and Egger’s tests were employed for detecting publication bias.

**RESULTS:**

Twelve articles, including 2 cohort studies and 10 case–control studies, and a total of 1210167 participants were identified. The pooled relative risk (RR) with 95% confidence interval (95% CI) implied that smoking was not associated with increased risk of meningioma in men and women combined (RR=1.09; 95% CI: 0.90–1.33). From the sex-stratified subgroup analysis, the risk of meningioma was significant in men (RR=1.42; 95% CI: 1.16–1.74). Risk of meningioma in women did not remain significant (RR=0.92; 95% CI: 0.73–1.16). There was a high heterogeneity in the results (I^2^=58.4%, p=0.002). Sensitivity analyses showed stable results and there was no evidence of publication bias.

**CONCLUSIONS:**

Cigarette smoking is not associated with a significantly increased risk of meningioma in the whole population, but there is a positive association in men but not in women.

## INTRODUCTION

Smoking is a risk factor for many diseases^1^. There are more than 7000 chemicals in tobacco smoke, hundreds of which are harmful. Smoking is popular all over the world. It is estimated that approximately 6 million people die each year from smoking and environmental tobacco exposure^2^. Some studies have found that smoking increases the risk of several types of cancer, including lung, oral, throat, esophageal, gastric, colon, and rectal cancer^3^. In addition, components such as n-nitroso compounds in cigarette smoke can cross the blood–brain barrier^4^. Animal experiments have proved that smoking is associated with meningioma^5-7^. Meningioma accounts for approximately 25% of all primary adult intracranial tumors, and it is more common in women. It is more common in middle-aged and elderly patients^8^. Many epidemiological studies have investigated the possible link between the occurrence of meningioma and smoking, but the results were inconsistent. Three studies found a positive association between active smoking (in men^9-11^) and meningioma. However, others studies did not find a positive association between active smoking (in men^12-14^, women^9,10,14-17^, or both^18,19^) and meningioma. Recently, a meta-analysis by Fan et al.^20^ concluded that smoking was not associated with a significantly increased risk of meningioma. We performed the present study to further investigate a possible association between active smoking and the risk of developing meningioma by sex-stratified analysis.

## METHODS

### Search strategy and selection criteria

According to the meta-analysis of observational studies in epidemiology (MOOSE) guidelines^21^, two authors searched the relevant publications in PubMed, Medline, Embase, and Science Direct. We restricted our literature search to human studies that were published in English and tried identifying non-published studies. Searching covered single words or combinations, including ‘meningioma’ or ‘meningeal neoplasms’ or ‘meningeal tumor’ with smoking (‘tobacco,’ ‘smoke,’ ‘cigarette’, ‘smoker’). To find more articles, a manual retrieval of relevant articles and references was performed. The inclusion criteria were: 1) the study assessed the relationship between smoking and meningioma; 2) a case-control study or cohort study; 3) the study reported relative risk (RR) or odds ratio (OR) and 95% confidence interval (CI), or the original data allowed this to be calculated; and 4) data of smoking status include smoking (including ever and current) versus never smoking. If the subject inhaled directly cigarettes that was regarded as active smoking. Active smokers were defined as active smoking of at least 100 cigarettes or for six months or more. Otherwise, subjects were classified as never active smokers. Criteria for exclusion were: 1) animal experiments or mechanistic research; 2) the study investigated passive smoking or environment smoking; and 3) the publication was in the form of a letter, conference paper, review, or case report.

### Data extraction and quality assessment

Two authors undertook independent evaluations of titles and abstracts of cited articles. The following data were extracted: first author’s name, publication year, study design, number of participants, country, assessment of outcome, estimated effect size (RR), corresponding 95% CI, and adjusted factors. Quality assessment was conducted using the Newcastle– Ottawa Quality Assessment Scale (NOS); studies with NOS score ≥7 were considered of high quality, and studies with NOS score ≥5 were considered of moderate quality^22^.

### Statistical analysis

Association analysis between smoking and meningioma was based on the adjusted RR and the 95% CI, as reported by eligible studies. The Q test and I^2^ statistic were used to assess heterogeneity among selected studies^23^. Considering the large variation in terms of study design and study population characteristics of all the included studies, it is more prudent to always use random-effects model regardless of the I^2^ value.

Subgroup analyses were conducted according to the following characteristics: geographical region (US/Europe or Asia), study design (case–control or cohort), sex (men or women), study quality (high or moderate), and adjustments of RR score (Yes or No).

Begg’s and Egger’s tests were employed for detecting publication bias. For evaluating the stabilities of the meta-estimates, sensitivity analysis was adopted by removing one article at a time. STATA version 13.0 was utilized for performing all data analyses.

## RESULTS

### Study selection

**Figure 1:**
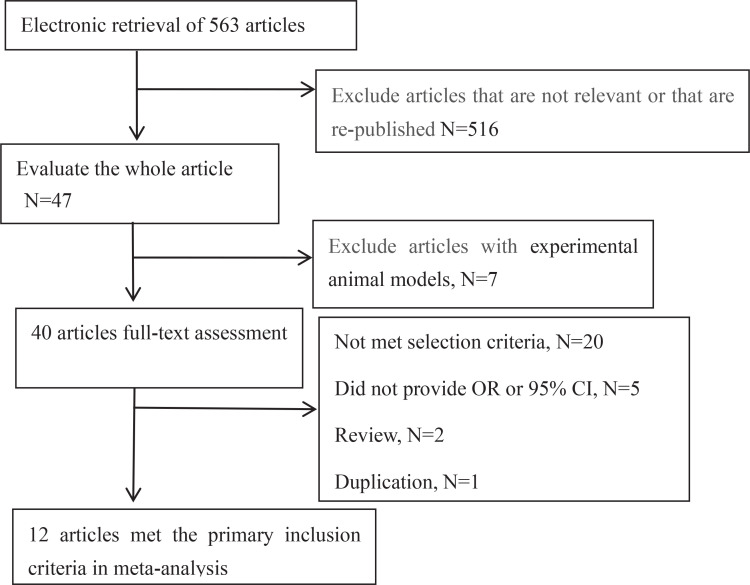
Flowchart presenting the steps of literature search and selection

In Figure 1, the details of the whole process and 12 eligible papers for meta-analysis are presented. The selected papers included 10 case–control studies^9-15,18,19,24^ and 2 cohort studies^16,17^. The selected studies were conducted in the US^9,11,13,15,17,24^, Canada^14,18^, Israel^10^, China^12^, UK^16^, and France^19^. Of the 12 selected articles, six reported smoking in men^9-14^ and eight in woman^9,10,12,14-17,24^. Studies on the different sexes were mostly conducted independently. According to the nine-point NOS, six studies^9,10,12,14,16,17^ were of high quality and six studies^11,13,15,18,19,24^ were of moderate quality. The details of each study are provided in Table 1.

**Table 1:** Main characteristics of included studies on the active smoking and risk of meningioma

*No.*	*Author, Year*	*Case Men/Women*	*Control Men/Women*	*Study design*	*Country*	*Sex*	*RR*	*95% CI*	*Adjustment*	*Score*
1	Phillips et al.^9^ 2005	57/143	114/286	Case-control	US	Men	2.10	1.05–4.20	Education	High
						Women	0.75	0.50–1.10		
2	Flint-Richter et al.^10^ 2011	71/171	84/196	Case-control	Israel	Men	2.13	1.09–4.16	Radiation	High
						Women	0.79	0.50–1.24		
3	Schildkraut et al.^11^ 2014	456/0	452/0	Case-control	US	Men	1.39	1.07–1.80	NA	Moderate
4	Hu et al.^12^ 1999	70/113	140/226	Case-control	China	Men	0.94	0.50–1.74	Income, education, occupational exposure to chemicals, consumption of fruit and vegetables	High
						Women	1.94	1.04–3.63		
5	Preston-Martin et al.^13^ 1989	70/0	70/0	Case-control	US	Men	1.21	0.60–2.46	NA	Moderate
6	Vida et al.^14^ 2014	26/67	317/331	Case-control	Canada	Men	1.47	0.56–3.90	Age, sex, education, region	High
						Women	0.99	0.51–1.92		
7	Preston-Martin et al.^15^ 1995	0/81	0/155	Case-control	US	Women	1.70	0.90–3.10	Age, menstruating, ERT use, OC, radiography	Moderate
8	Benson et al.^16^ 2008	0/372	0/1177087	Cohort	UK	Women	0.86	0.67–1.10	Height, BMI, strenuous exercise, socioeconomic level, alcohol intake, parity, age at first birth, OC	High
9	Johnson et al.^17^ 2011	0/125	0/27791	Cohort	US	Women	0.90	0.60–1.33	Education, residence, alcohol use, physical activity index	High
10	Choi et al.^18^ 1970	23	23	Case-control	Canada	Both	0.59	0.18–1.90	NA	Moderate
11	Allès et al.^19^ 2016	193	392	Case-control	France	Both	1.31	0.86–2.00	NA	Moderate
12	Lee et al.^24^ 2006	0/217	0/248	Case-control	US	Women	0.60	0.40–0.90	NA	Moderate

OC: oral contraceptive. ERT: estrogen replacement therapy. NA: not applicable. BMI: body mass index.

### Cigarette smoking and risk of meningioma

**Figure 2:**
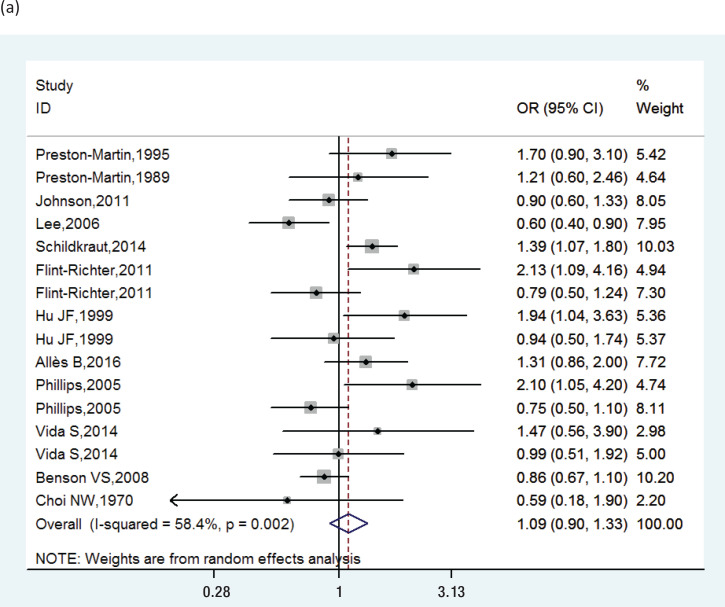

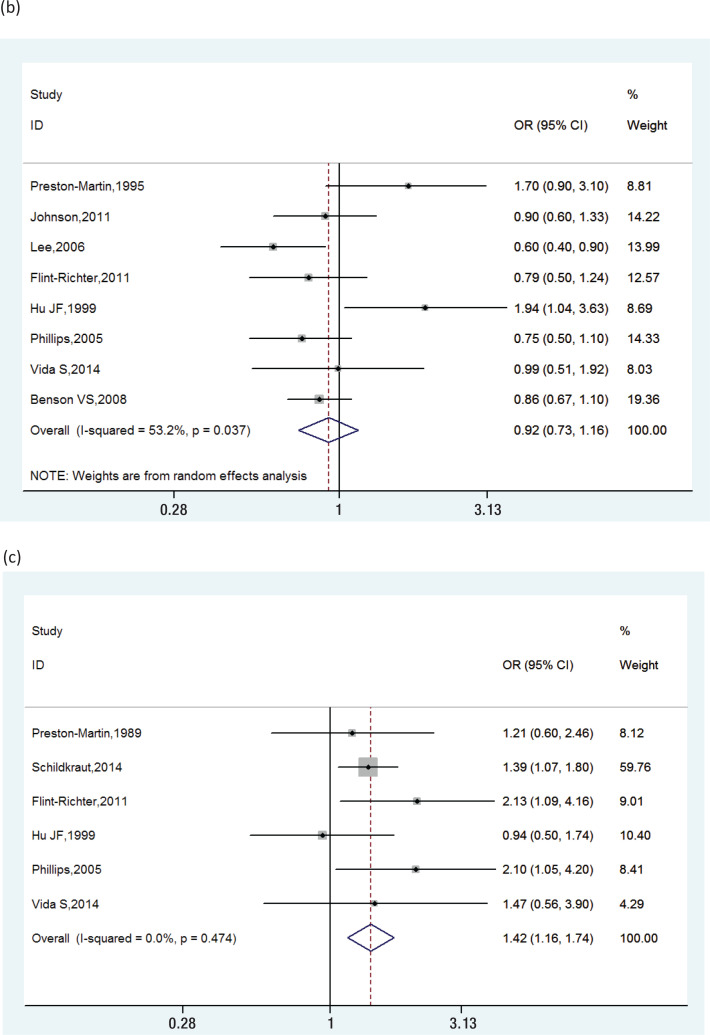
Forest plots showing individual and pooled RRs with 95% CI of the risk between active smoking and meningioma: (a) in men and women combined, (b) in women, (c) in men

The pooled RRs of cigarette smoking with meningioma are shown in Figure 2. We found significant heterogeneity (I^2^=58.4%), and a random-effects model was used to calculate the pooled RR. The combined RR was 1.09 (95% CI: 0.90–1.33).

### Subgroup and sensitivity analysis

Subgroup analysis on the basis of study design, geographical regions, publication year, and adjustments of RR score showed that the results remained similar. In women, smoking was not a significant risk for meningioma (RR=0.92; 95% CI: 0.73–1.16) among 8 studies (I^2^=53.2%, p=0.037). However, in men, smoking was a significant risk for meningioma (RR=1.42; 95% CI: 1.16–1.74), within six studies (I^2^=0.0%, p= 0.474). Sensitivity analysis confirmed that the results were stable by the removal of one study at a time. Table 2 and Supplementary file Figure 1 show the data from our subgroup and sensitivity analyses.

**Table 2:** Subgroup and sensitivity analysis of all the included studies

*Subgroup*	*Number of reports*	*RR (95% CI)*	p*	*I ^2^ (%)*
**Geographical region**				
US/Europe	10	1.03 (0.91–1.16)	0.124	44.8
Asia	2	1.20 (0.91–1.60)	0.044	53.7
**Study design**				
Case–control	10	1.13 (0.99–1.29)	0.002	60.2
Cohort	2	0.87 (0.71–1.07)	0.849	0.00
**Sex**				
Men	6	**1.42 (1.16–1.74)**	0.474	0.00
Women	8	0.92 (0.73–1.16)	0.037	53.2
**Study quality**				
High	6	1.09 (0.93–1.29)	0.024	52.9
Moderate	6	1.02 (0.87–1.18)	0.004	71.4
**Adjustments of RR score**				
Yes	7	1.01 (0.88–1.17)	0.011	56.3
No	5	1.12 (0.93–1.35)	0.009	70.3

* For heterogeneity test.

### Publication bias

A funnel plot was employed to evaluate publication bias. There was no obvious publication bias. Begg’s and Egger’s tests yielded no statistical significance (p=0.192 and p=0.360, respectively, Supplementary file Figure 2).

## DISCUSSION

Meningioma is the most common subtype of brain tumor in adults, with an incidence rate of 3.5 per 100000 person-years^25^. The 5-year survival rate is 72% for women and 66% for men^26^. These tumors are most common in women, with a women-to-men ratio of about 2:1, and most commonly occur between adolescence and menopause. Meningiomas are tumors that originate in the arachnoid layer of the meninges. Although generally benign in histological appearance and behavior, 5–10% of these tumors are malignant. At present, the cause of meningioma is still largely unclear, but several studies have shown that the triggers for their development include radiation, brain injuries, smoking, and female hormones^9,27^. Cigarette smoke is a complex mixture of chemicals and is the single most important cause of cancer in humans. It has been shown to induce tumors in many organs and tissues.

This is the largest meta-analysis to examine the relationship between cigarette smoking and meningioma risk. A total of 1210167 participants were included. According to our study, active smoking may increase the risk of meningioma in men (RR=1.42; 95% CI: 1.16–1.74), but not significantly in the whole population (RR= 1.09; 95% CI: 0.90–1.33). Our results are similar to those of another meta-analysis^20^ of smoking and risk of meningioma, which obtained an OR of 0.95 (95% CI: 0.87–1.07). The study by Fan et al.^20^ included 9 papers and passive smoking, whereas 12 papers were included in the present study. Five studies^11,14,15,17,19^ which were not included in the Fan et al.^20^ study were included in our meta-analysis as they met our inclusion criteria. Another meta-analysis obtained an OR for smoking of 0.82 (95% CI: 0.68–0.98, n=6) for women and 1.39 (95% CI: 1.08–1.79, n=5) for men^28^. Our results show that smoking is not a significant risk for women (RR=0.92; 95% CI: 0.73–1.16, n=8), but it plays a bigger role in men (RR=1.42; 95% CI: 1.16–1.74, n=6). There are three possible reasons to explain this difference. Firstly, our study included a larger sample size and passive smoking was excluded. Secondly, the exposure intensity is different, and active smoking is much stronger. Lastly, the type of tobacco is different between men and women, and men smokers smoke more than women smokers^29^.

### Limitations

There are still some limitations in this study. First, there was a lack of accurate assessment of exposure to cigarette smoking. Despite a feasibility of crude classifications, this was inevitable. Second, the studies used questionnaires to evaluate smoking, but self-reported methods could easily result in reporting bias. Researchers should use biomarkers or specific substrates in the body to determine exposure doses more accurately. Third, we did not study the relationship between different levels of tobacco exposure and the risk for meningioma because there was insufficient information about the dose–response relationship.

## CONCLUSIONS

This meta-analysis indicates that cigarette smoking does not increase the risk of developing meningioma, in the whole population. However, sex-stratified subgroup analysis indicates a positive association in men but not in women.
