# Supplementary material for: A meta-analysis of active smoking and risk of meningioma
Source: Tob Induc Dis. 2021 May 10;19:34. doi: 10.18332/tid/133704 (PMC8106389; doi:10.18332/tid/133704)
Supplement: Supplementary file 1 [file TID-19-34-s1.pdf]

Supplementary Figure 1. The result of sensitivity analysis of included studies on the active smoking and risk of meningioma

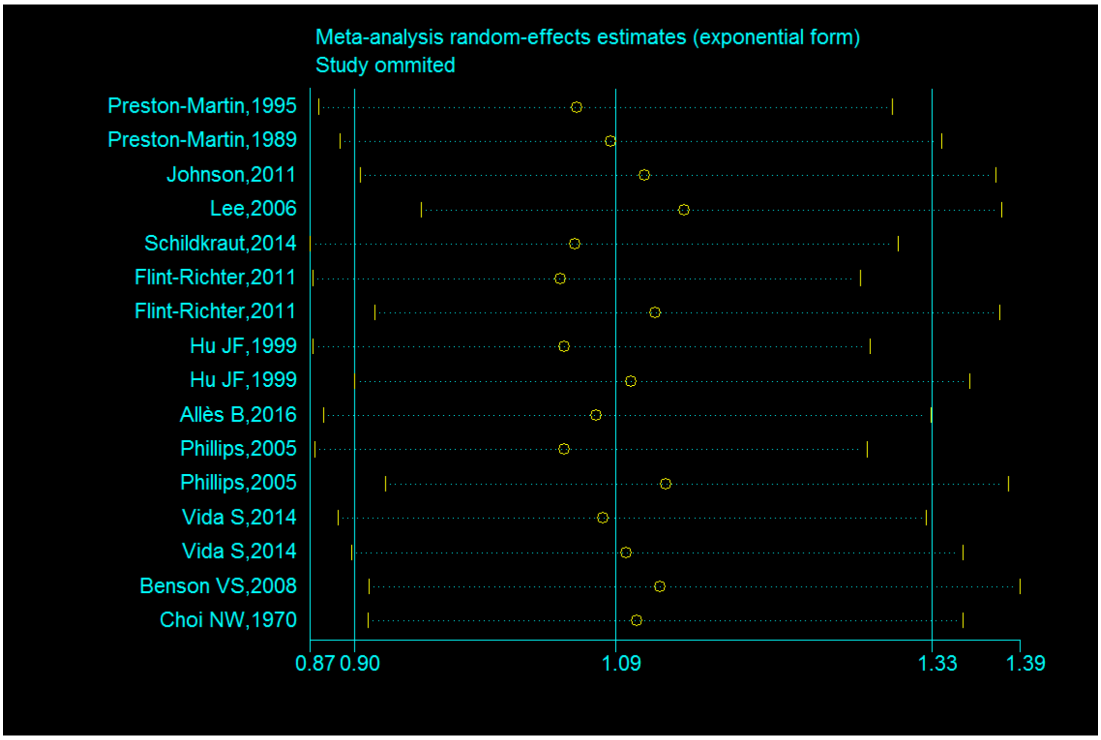

Supplementary Figure 2. The funnel plot of meta-analysis to evaluate publication bias.

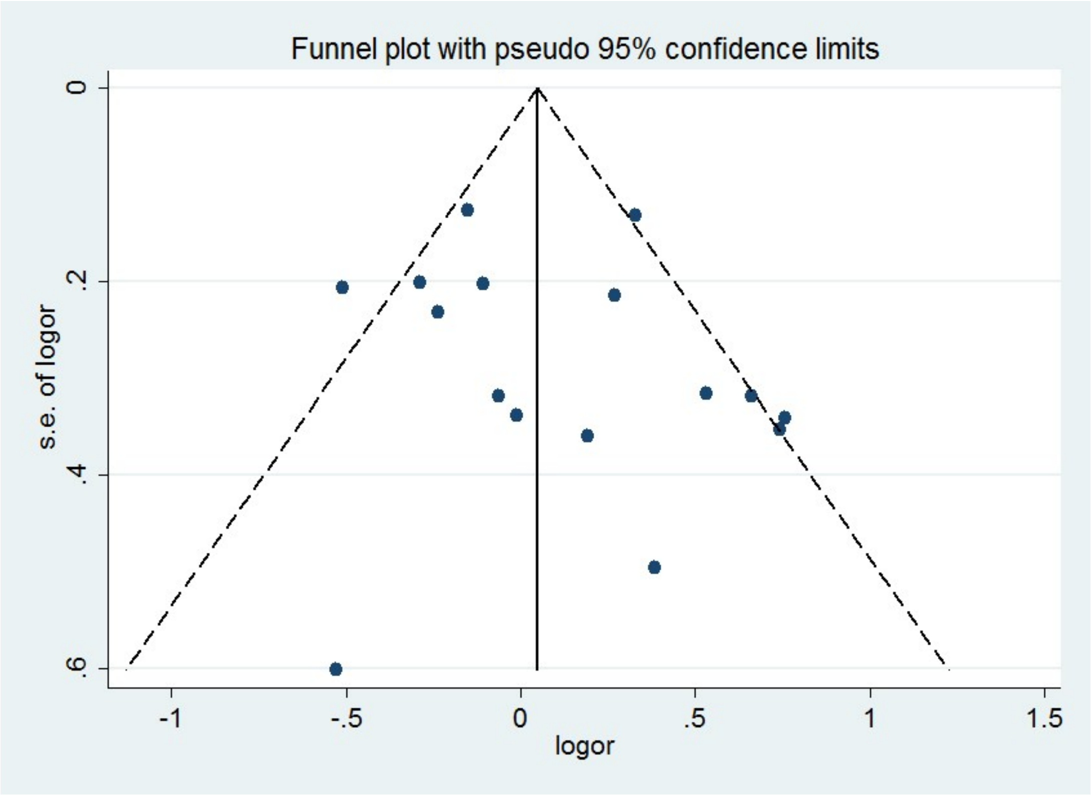

©2021 Chao H. et al.
